# The role of default mode network in semantic cue integration

**DOI:** 10.1016/j.neuroimage.2020.117019

**Published:** 2020-10-01

**Authors:** Lucilla Lanzoni, Daniela Ravasio, Hannah Thompson, Deniz Vatansever, Daniel Margulies, Jonathan Smallwood, Elizabeth Jefferies

**Affiliations:** a Department of Psychology, University of York, UK; b Department of Psychological Sciences, University of Bergamo, Italy; c School of Psychology, University of Surrey, UK; d Institute of Science and Technology for Brain-inspired Intelligence, Fudan University, Shanghai, PR China; e Institute Du Cerveau et de la Moelle épiniére (ICM), Paris, France

**Keywords:** Default mode, Integration, Principal gradient, Semantics, Cueing

## Abstract

Recent accounts of large-scale cortical organisation suggest that the default mode network (DMN) is positioned at the top of a principal gradient, reflecting the separation between heteromodal and unimodal sensory-motor regions in patterns of connectivity and in geodesic distance along the cortical surface (Margulies et al., 2016). This isolation of DMN from external inputs might allow the integration of disparate sources of information that can constrain subsequent cognition. We tested this hypothesis by manipulating the degree to which semantic decisions for ambiguous words (e.g. jam) were constrained by preceding visual cues depicting relevant spatial contexts (e.g. supermarket or road) and/or facial emotions (e.g. happy vs. frustrated). We contrasted (i) the effects of a single preceding cue with a no-cue condition employing scrambled images, and (ii) convergent spatial and emotion cues with single cues. Single cues elicited stronger activation in the multiple demand network relative to no cues, consistent with the requirement to maintain information in working memory. The availability of two convergent cues elicited stronger activation within DMN regions (bilateral angular gyrus, middle temporal gyrus, medial prefrontal cortex, and posterior cingulate), even though behavioural performance was unchanged by cueing – consequently task difficulty is unlikely to account for the observed differences in brain activation. A regions-of-interest analysis along the unimodal-to-heteromodal principal gradient revealed maximal activation for the convergent cue condition at the heteromodal end, corresponding to the DMN. Our findings are consistent with the view that regions of DMN support states of information integration that constrain ongoing cognition and provide a framework for understanding the location of these effects at the heteromodal end of the principal gradient.

## 1 Introduction

The context in which we encounter concepts in our daily life influences the manner in which we think about them. Hearing the word *jam* at the kitchen table, for example, one might activate a number of concepts related to food, its taste and emotional valence. The same word *jam* on the traffic news, however, might bring up very different thoughts and emotions. Although studies have manipulated sentence contexts to constrain the interpretation of ambiguous words (e.g. Noonan et al., 2010; Rodd et al., 2016; Rodd et al., 2005; Rodd et al., 2004; Rodd et al., 2013; Vitello and Rodd, 2015), cues beyond language have rarely been employed (for an exception see Lanzoni et al., 2019). Consequently, relatively little is known about how non-verbal cues, such as spatial location and affect, constrain meaning retrieval or the neural mechanisms that underlie this effect. The current study addressed this issue by manipulating the availability of spatial and facial emotion cues prior to semantic decisions about ambiguous words.

Contemporary models of semantic cognition suggest that retrieval is supported by a dynamic interplay of conceptual knowledge with retrieval processes (Hoffman et al., 2018; Jefferies, 2013; Lambon Ralph et al., 2016). Conceptual representations are rich and comprise features from multiple sensory modalities (e.g. an apple is a *sweet* fruit, with a *rounded* shape and a *smooth hard* surface which is often *red*, *yellow* or *green*). According to the Hub and Spoke model of conceptual representation, the ventrolateral anterior temporal lobe (ATL) ‘hub’ integrates features encoded in sensory-motor cortical ‘spokes’ to generate coherent representations – e.g. our concept ‘apple’ (Chiou and Lambon Ralph, 2019; Patterson et al., 2007; Lambon Ralph et al., 2016). However, hub and spoke representations are not sufficient to support *flexible* semantic cognition; we also dynamically vary the aspects of knowledge that we retrieve about concepts depending on the context. Semantic processing may draw on different large-scale networks depending on whether retrieval is usefully constrained or miscued by the context.

In line with this view, semantic sites have been shown to overlap with distinct large-scale networks that are recruited differentially depending on the task demands. When non-dominant associations are required by a task, or the prior context is unhelpful, a ‘semantic control network’ is recruited (including left inferior frontal gyrus and posterior middle temporal gyrus), which may shape retrieval to suit the circumstances (Badre and Wagner, 2005, 2006; Davey et al., 2016; Hallam et al., 2016; Krieger-Redwood et al., 2015; Noonan et al., 2013; Whitney et al., 2011). In contrast, other key sites for semantic cognition, such as lateral ATL and angular gyrus (AG), have patterns of intrinsic connectivity that are partially overlapping with aspects of the Default Mode Network (DMN) (Davey et al., 2016; Humphreys and Lambon Ralph, 2014; Jackson et al., 2016; Seghier et al., 2010). The role of DMN regions in semantic cognition remains controversial: a meta-analysis by Binder et al. (2009) found peak activation for semantic tasks in AG, while other researchers have characterized AG as a task-negative region which deactivates across semantic and non-semantic tasks (Humphreys et al., 2015; Humphreys and Lambon Ralph, 2014; Mollo et al., 2017). DMN regions, including AG, typically show anti-correlation with task-positive regions within the multiple demand network (MDN; Blank et al., 2014; Davey et al., 2016; Fox et al., 2005). Nevertheless, TMS studies have shown that AG plays a critical role in the efficient retrieval of dominant aspects of knowledge (Davey et al., 2015). There are also demonstrations of a role for the DMN in semantic retrieval even when tasks are relatively hard. For example, Murphy et al. (2018) found greater DMN recruitment both when participants made judgements based on their memory of preceding trials (as opposed to stimuli present on the screen), and when the decisions involved semantic categories as opposed to perceptual features.

Recent studies have suggested that semantic regions allied to DMN, including AG, support the combination of concepts into meaningful and more complex representations (e.g. Price et al., 2015; for a review see Pylkkänen, 2019). These regions show a stronger response when coherent conceptual combinations or heteromodal features are presented (Bemis and Pylkkänen, 2011; Price et al., 2015, 2016; Pylkkänen, 2019; Teige et al., 2018, 2019). The suggested critical role of the DMN in conceptual integration fits well with the observation that the DMN lies at the top of a cortical hierarchy. Through decomposition of resting-state connectivity, Margulies et al. (2016) identified a principal gradient of macroscale organization, anchored at one end by sensory regions and at the other end by heteromodal cortex, corresponding to the DMN. This separation of DMN from unimodal cortex in intrinsic connectivity relates to geodesic distance – DMN sites are located relatively far away from primary sensory-motor cortex along the cortical surface (Margulies et al., 2016). Greater distance along the gradient might allow the brain to support forms of cognition that rely on memory, as opposed to information in the external environment (Murphy et al., 2019). Distance might also support increasing levels of abstraction from sensory-motor features, allowing the formation of heteromodal conceptual representations from the integration of these diverse sources of information (Buckner and Krienen, 2013; Mesulam, 1998; Patterson et al., 2007; Smallwood, 2013). In line with this idea, default mode regions might show a greater response in semantic tasks when multiple aspects of a concept are activated during retrieval.

In the present study, we tested the view that semantically-relevant regions within the DMN, in particular AG, contribute to conceptual integration. We adopted a paradigm recently developed to assess the impact of non-verbal cues in patients with semantic aphasia, who have deficits of semantic control (Lanzoni et al., 2019). Participants were shown 0, 1 or 2 cues that were relevant to the subsequent interpretation of an ambiguous word: they saw photographs of spatial contexts, facial emotions or scrambled meaningless versions of these cues. The cues alone were not sufficient to prime the concepts and did not influence behavioural performance (for example, supermarket and happy face can be linked in many ways and do not strongly anticipate jam as food). Nevertheless, the cues allowed the subsequent semantic decisions to unfold in a conceptually-rich context. If semantic integration occurs in the DMN, comparing semantic decisions in the context of multiple convergent cues as opposed to single cues should reveal increased activation within this network and in particular in AG – even though semantic decisions to ambiguous words are relatively cognitively effortful. In contrast, brain regions that selectively encode and maintain semantic cue information *prior* to integration should be spatially distinct from DMN: the neural basis of cue maintenance might be maximally revealed by a contrast of single cue over no cue trials (as this contrasts situations where there are working memory demands versus no requirement to maintain information). MDN is a candidate network for attentional and working memory components of the cueing task, since this network is associated with executively demanding aspects of cognition, including working memory and the maintenance of task rules, across domains (e.g. Owen et al., 2005; Naghavi and Nyberg, 2005; Dosenbach et al., 2006). For example, a study by Dumontheil et al. (2011) found activation in several parts of MDN during the presentation of task instructions, which might reflect the creation of a task-model or framework for ongoing cognition.

Additionally, we predicted that the effect of conceptual integration but not cue load would be located at the heteromodal end of the principal gradient (Margulies et al., 2016), providing a framework for understanding *why* information integration effects occur where they do within the cortex: these effects should be greatest at the DMN apex of the gradient, which is maximally separated (both in terms of physical distance and in connectivity terms) from unimodal input or ‘spoke’ regions associated with processing specific features. In contrast to our standard whole-brain cluster-corrected contrasts, the focus of this analysis was not on the functional contribution of specific regions, such as AG, to cue integration, but instead on whole-brain patterns that include similar functional transitions between heteromodal and unimodal cortex in distant cortical regions.

## 2 Materials and methods

### 2.1 Participants

Twenty-seven healthy right-handed native English-speaking participants with normal or corrected-to-normal vision were recruited from the University of York (9 males, mean age 21.5, SD 2.9, range 19–30). Participants received monetary compensation or course credits. One dataset was excluded due to technical problems that resulted in no behavioural responses being recorded, leaving 26 subjects in the final sample. In a subsequent analysis we examined resting-state fMRI data from 86 participants (22 males; mean age 20.3, range 18–32 years), twelve of whom were also in the main sample. The research was approved by the York Neuroimaging Centre Ethics Committee and participants provided written informed consent.

### 2.2 Materials

The cueing paradigm, adapted from Lanzoni et al. (2019), presented pictures of facial expressions and spatial locations prior to semantic judgements about ambiguous words. The stimuli are available on the Open Science Framework (https://osf.io/wp6a7/).^1^ Thirty English homonyms were selected from the Free Association Norms of Twilley et al. (1994), and the Gawlick-Grender & Woltz norms (1994). We chose items where the different interpretations were associated with different facial expressions (e.g. jam with *traffic* is associated with frustration while jam with *strawberry* is associated with pleasure). We also chose items where different interpretations were associated with different locations (e.g. a motorway for *traffic* jam and a supermarket for *strawberry* jam). We then generated four target words for each probe, two for each interpretation. This resulted in 120 probe-target pairs. For instance, the probe jam appeared in four trials, twice paired with a target referring to *traffic* (jam-horn or jam-delay) and twice paired with a target referring to the alternative interpretation (jam-spoon or jam-bread). Although we did not manipulate the difference in frequency between the two alternative meanings, one interpretation of the homonym was dominant over the other (i.e., a larger proportion of subjects generated words linked to that interpretation, as reported in Twilley et al., 1994). Dominance was controlled by counterbalancing the assignment of each interpretation to the different experimental conditions across participants. For each combination of probes and targets, two unrelated distractors were selected. Latent Semantic Analysis (as implemented in lsa.colorado.edu) was used to calculate the similarity in semantic space between the probe and the targets vs. probe and distractors (parameters used: space – General reading up to 1st year college, comparison type - term to term, number of factors – maximum). This confirmed that the strength of the relationship between probe and distractor (M ​= ​0.08, SD ​= ​0.04) was significantly weaker compared to the association between probe and target (M ​= ​0.22, SD ​= ​0.10; t (29) ​= ​7.17, p ​< ​.001). Distractors and target words were matched for lexical frequency (SUBTLEX-UK database, van Heuven et al., 2014; t ​= ​0.89, p ​= ​.380), word length (t ​= ​−1.44, p ​= ​.154), and concreteness (Brysbaert et al., 2014; t ​= ​0.58, p ​= ​.564).

Pictures of facial emotional expressions and spatial locations were used to prime the relevant meaning of the homonym. Each picture was used only once across the entire experiment, making it impossible for participants to predict the following probe word on the basis of the cue. Images of facial expressions were chosen from the Radboud Faces Database (Langner et al., 2010) and included eight basic emotions: happy, angry, sad, disgusted, contemptuous, surprised, neutral, fearful. In selecting the affect cues we ensured that the same face from the Radboud Database would not be presented in the same emotional expression in other trials. Therefore, for trials that required the same emotional expression we chose different actors. Pictures of spatial contexts were downloaded from Google images.

**Fig. 1:**
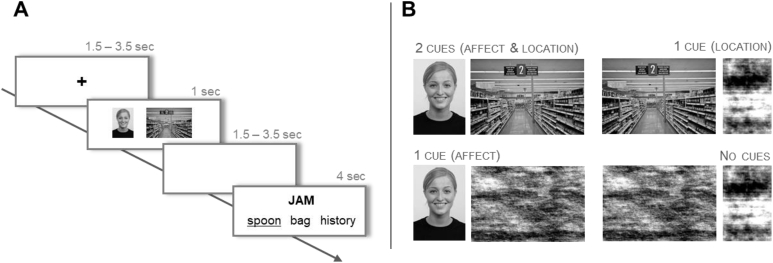
A. After an initial fixation cross (1500–3000 ​ms), participants were presented with cue images for 1000 ​ms, before moving to a blank screen (1500–3000 ​ms). Following that, a probe word was presented above a target and two unrelated distracters, triggering the onset of the decision-making period. The probe and choices remained visible for a fixed interval of 4000 ​ms. B. The four levels of the variable cue are shown.

The emotion and location cues could appear together in the same trial (2 cues condition), they could be presented alone (1 cue affect or location conditions), in which case they were paired with one meaningless scrambled image, or two scrambled images were provided (no-cue condition). Images were converted to greyscale, matched for luminance and scrambled using the SHINE toolbox (Willenbockel et al., 2010). Images were also brought to a fixed dimension (600 ​× ​400 pixels for location and 260 ​× ​400 for affect cues) using Matlab (The MathWorks Inc., Natick, MA, US). Fig. 1B shows the 4 cue conditions, which were used to examine three levels of constraint on semantic retrieval. The location of the emotion and location cues (to the left or right of the screen) was counterbalanced within each run. Finally, to ensure that people could not make their decisions based only on the cue, in each trial one of the distractors was related to either the emotional cue or the visuo-spatial cue presented before the semantic task (in Fig. 1A, the distractor ‘bag’ is related to the location cue – supermarket). The assignment of the emotion-related and location-related distractors to the different conditions was counterbalanced within participants, such that each probe appeared twice with an emotion-related distractor and twice with a location-related distractor.

### 2.3 Procedure

The MRI session included a high-resolution structural scan, a FLAIR sequence and four functional runs of approximately 9 ​min each. Each trial started with a fixation cross of random duration between 1500 and 3000 ​ms (Fig. 1A). Two cue pictures or scrambled cues were then presented for 1s, followed by another jittered inter-stimulus interval (ISI: 1500–3000 ​ms). Participants were asked to pay attention to the cues, and they were told that these would be helpful images on some trials, and meaningless images on other trials. Next, four words appeared on screen – a probe word at the top and three response options underneath, marking the start of the semantic task. Participants were asked to decide which of the three options had the strongest semantic relationship to the probe, and they were encouraged to make the semantic decision based on the words and not on the previously seen images. Although the time to respond was fixed (4s), participants were asked to respond as quickly and accurately as possible. Each of the 30 probes was presented once within each run, resulting in 30 semantic trials. The order of presentation was randomized and stimuli were counterbalanced so that, across all participants, each probe-target combination appeared in all four cue conditions. Each run had a total of eight non-semantic trials, in which words were replaced with strings of the letter ‘X’ matched in length to the words. Here the task was to press any key. The scrambled images used in non-semantic trials were created equally often from face and location photos. Two null trials were also included to improve task modelling. During null trials participants saw a blank screen for the same duration of 4 ​s.

### 2.4 fMRI acquisition

Whole brain fMRI data acquisition was performed using a GE 3 ​T HDx Excite MRI scanner. Structural MRI data acquisition in all participants was based on a T1-weighted 3D fast spoiled gradient echo sequence (TR ​= ​7.8 ​ms, TE ​= ​minimum full, flip-angle ​= ​20°, matrix size ​= ​256 ​× ​256, 176 slices, voxel size ​= ​1.13 ​× ​1.13 ​× ​1 ​mm). A gradient-echo EPI sequence was used to collect functional data from 60 interleaved bottom-up axial slices aligned with the temporal lobe (TR ​= ​3s, TE ​= ​18.9 ​ms, FOV ​= ​192 ​× ​192 ​× ​180 mm, matrix size ​= ​64 ​× ​64, slice thickness ​= ​3 ​mm, slice-gap ​= ​3 ​mm, voxel size ​= ​3 ​× ​3x3 mm^3^, flip-angle ​= ​90°). An intermediary FLAIR scan with the same orientation as the functional scans was collected to improve the co-registration between subject-specific structural and functional scans.

### 2.5 Data preprocessing

#### 2.5.1 Behavioural pre-processing and analysis

We examined accuracy, median response time (RT), RT variability and response efficiency in separate repeated-measures ANOVAs to characterize differences in performance across the 4 semantic conditions (0 cues, 1 cue affect, 1 cue location, 2 cues: affect and location). One keypress was not recorded for two participants and these missing RT values were replaced with the group median for that condition. Response efficiency scores were used to account for any speed-accuracy trade-offs: the median RT for correct responses for each subject in each condition was divided by the mean accuracy in the same condition (Townsend and Ashby, 1983). We also examined trial-to-trial variability, using the standard deviation of RT for each participant in each condition.

#### 2.5.2 MRI data pre-processing

FMRI data processing was carried out using FEAT (FMRI Expert Analysis Tool) Version 6.0, part of FSL (FMRIB’s Software Library, www.fmrib.ox.ac.uk/fsl). Registration of the high resolution structural to standard space (Montreal Neurological Institute – MNI) was carried out using FLIRT (Jenkinson et al., 2002; Jenkinson and Smith, 2001). Pre-processing of the functional image included motion correction using MCFLIRT (Jenkinson et al., 2002), slice-timing correction using Fourier-space time-series phase-shifting (interleaved), non-brain removal using BET (Smith, 2002), spatial smoothing using a Gaussian kernel of FWHM 5 ​mm, grand-mean intensity normalisation of the entire 4D dataset by a single multiplicative factor, and high-pass temporal filtering (Gaussian-weighted least-squares straight line fitting, with sigma ​= ​50.0s).

### 2.6 Statistical modelling

Pre-processed time series were modelled using a general linear model using FILM correcting for local autocorrelation (Woolrich et al., 2001). We used an event-related design. We built two separate models, a *semantic decision model* to look for brain changes during semantic decisions following different levels of cueing, and a *cue model* to identify brain regions that responded to the presentation of the cues. Our key focus was on the semantic decision model, since this established whether specific networks or gradient patterns were associated with making semantic decisions in the context of single or convergent cues. The *semantic decision model* included 8 ​EVs: correct semantic decisions following each of the 4 experimental conditions (0 cues, 1 cue affect, 1 cue location, 2 cues), non-semantic trials where strings of “Xs” were presented, remaining time in the semantic trials after making a decision before the start of a new trial, cue presentation period (combining all the cue presentation events, irrespective of the cue condition), and incorrect semantic trials. Given that this model revealed two distinct networks associated with the maintenance of single cues as opposed to no cues, and the convergence of multiple cues vs. a single cue, we then elected to examine the response during cue presentation in a second stage of the analysis. The *cue model* included 6 Explanatory Variables (EVs) corresponding to the 4 cue conditions (0 cue condition containing scrambled images, 1 face cue ​+ ​scrambled image, 1 location cue ​+ ​scrambled image, 2 cues: face and location), the semantic task, and the non-semantic task. The cue model established whether MDN regions responding to one ​> ​no cues showed load-dependent effects during cue encoding, consistent with increasing working memory demands of cue maintenance. However, it is important to acknowledge that the study was not designed to examine the cue phase in this fashion, and there are limitations of this exploratory analysis – in particular, the study did not de-confound the order of the cue presentation and semantic decision phases, as cues were always followed by the semantic task (albeit separated by a jittered interval; see limitations in Discussion). All regressors were modelled using a variable epoch model, with the appearance of the words (or the cue images, for the *cue model*) as the start of the event and the response time (or the duration of the cue presentation) as the duration of the event. Convolution of the hemodynamic response was achieved using a Gamma function (phase ​= ​0, SD ​= ​3, mean ​= ​6). Temporal derivatives were added to each regressor. Nuisance regressors included standard ​+ ​extended motion parameters. Absolute framewise displacement ranged from 0.05 ​mm to 0.64, with a mean value of 0.21 ​mm across the 4 runs.

We then averaged contrast estimates over the four runs within each subject using a fixed effects model, by forcing the random effects variance to zero in FLAME (FMRIB’s Local Analysis of Mixed Effects) (Beckmann et al., 2003; Woolrich, 2008; Woolrich et al., 2004). The group analysis was carried out using FLAME (FMRIB’s Local Analysis of Mixed Effects) stage 1 (Beckmann et al., 2003; Woolrich, 2008; Woolrich et al., 2004). Z (Gaussianised T/F) statistic images were thresholded using clusters determined by z ​> ​3.1 and a (corrected) cluster significance threshold of p ​= ​.05 (Worsley, 2001). Our analysis focused on the comparison between semantic decisions which followed different levels of cue: 2 cues >1 cue (collapsing across emotion and location cues) and 1 cue ​> ​0 cues.

Cognitive decoding of the main contrasts of interest was performed in Neurosynth, an automated meta-analysis tool (Yarkoni et al., 2011). Unthresholded z maps were uploaded to Neurosynth to obtain psychological terms associated with the patterns of activation in our results. Where multiple terms had the same meaning (e.g. default, default mode, DMN, network DMN, default network), only the word with the highest correlation value was retained. This analysis provides additional evidence about the functional role of the regions within different maps, by comparing the results to previous studies which have reported similar patterns of activation.

Finally, we wanted to examine whether the observed pattern of BOLD response in DMN regions reflected the macroscale cortical organization captured by the principal gradient (Margulies et al., 2016). In line with previous studies by our group (Murphy et al., 2018, 2019), this analysis leverages the explanatory power of the unimodal to heteromodal gradient to account for differences between experimental conditions. Consistently with our predictions of greater DMN recruitment during information integration, we expected to observe a higher response in regions towards the heteromodal end of the gradient in the 2 ​> ​1 contrast. Decile bins along the gradient were calculated using the methods outlined by Margulies et al. (2016). The original gradient map provided values from 0 to 100 for each voxel in the brain (0 ​= ​unimodal end; 100 ​= ​DMN). This map was then divided into ten-percentile bins: all voxels with values 0–10 were assigned to bin1; voxels with values 11–20 to bin 2, etc., yielding 10 bins in total. The total number of voxels in each bin was near-identical (each contained 6133 to 6135 voxels). This analysis provides unique insights by focusing on whole-brain patterns associated with particular aspects of cued semantic retrieval, as opposed to the role of specific brain regions. The analysis can establish whether peaks associated with cue integration across the cortex are located at the apex of the gradient from heteromodal to unimodal processing, in line with the expectation that heteromodal cortex supports information convergence.

## 3 Results

### 3.1 Behavioural results

A repeated measures ANOVA examining response efficiency revealed no significant differences across conditions [F(3,75) ​= ​0.62, p ​= ​.605, *η*^2^ ​= ​0.02], indicating that semantic decisions following two cues were not easier than trials with less contextual support (one cue or no cue). The means and standard error for each condition are provided in Figure S1 and Table S1 (Supplementary Materials). There were also no significant differences between conditions in accuracy [F(3,75) ​= ​0.14, p ​= ​.939, *η*^2^ ​= ​0.01], median response time [F(3,75) ​= ​0.95, p ​= ​.420, *η*^2^ ​= ​0.04] or response time variability [F(3,75) ​= ​1.26, p ​= ​.296, *η*^2^ ​= ​0.05]. All statistical values are provided in Table S2.

### 3.2 fMRI results

First, we report the whole-brain univariate results for models examining (i) how the BOLD response during semantic decision-making changes as a consequence of cues (semantic decision model) and (ii) the response to cue presentation (cue model). The coordinates for cluster peaks are reported in Table S3 (Supplementary Materials) and statistical maps are available in Neurovault (https://neurovault.org/collections/6198/). Next, to test one account of the response to single cues vs. no cues during semantic decision-making, we present a region of interest (ROI) analysis examining the response to different numbers of cues during cue presentation, in regions defined by the semantic decision model. This exploratory analysis establishes whether these regions behave in a load-dependent manner during cue encoding. Finally, we examine whether integration effects in DMN regions are captured by a macroscale gradient of cortical organization, using a series of ROIs positioned from the heteromodal to the unimodal end of this gradient. Figures were created using BrainNet Viewer (Xia et a., 2013; http://www.nitrc.org/projects/bnv/) and Surf Ice (https://www.nitrc.org/projects/surfice/).

#### 3.2.1 Whole-brain results

##### 3.2.1.1 Semantic decision model

**Fig. 2:**
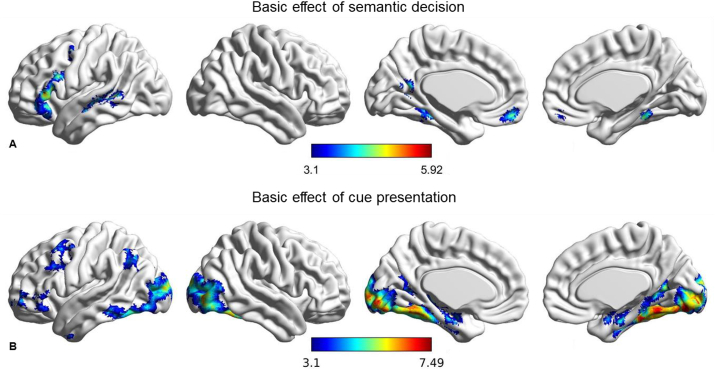
A. Basic effect of uncued semantic decision (semantic no cue ​> ​letter strings at decision time period). B. Basic effect of cue presentation (2 cues ​+ ​1 cue ​> ​0 cue at cue time period). Coordinates of cluster peaks for these basic comparisons are reported in Table S3.

**Fig. 3:**
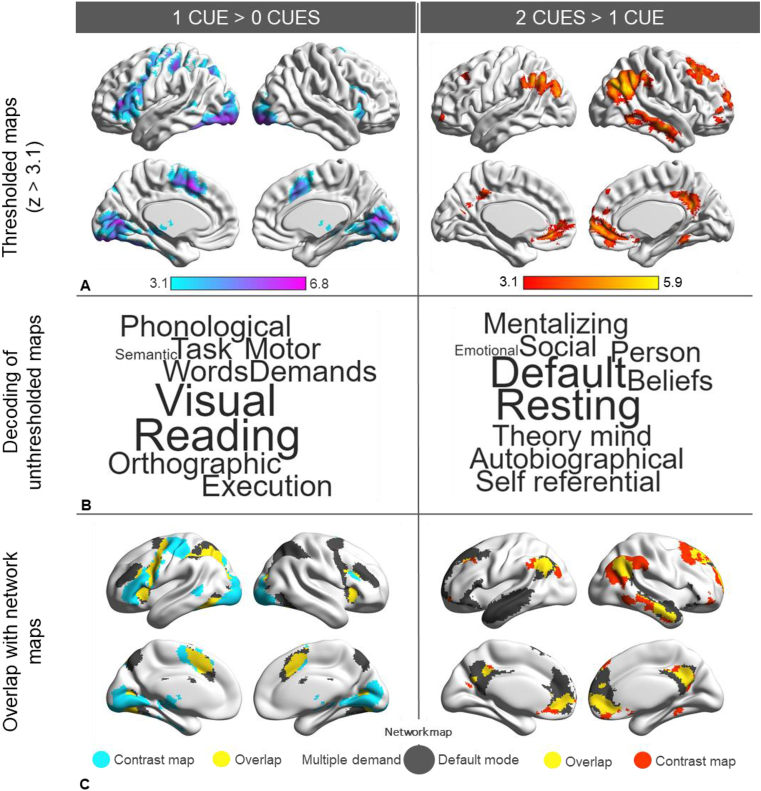
Results for the main contrasts of interest in the semantic decision model: the left side of the figure contains results for 1 cue >0 cues, while the 2 cues >1 cue contrast is shown on the right. A. Contrast maps thresholded at z ​> ​3.1. B. Word clouds produced by plotting the top 10 terms positively associated with the contrast map. C. Overlap of the 1 ​> ​0 contrast with the multiple demand network (Duncan et al., 2010) and the contrast of 2 ​> ​1 with the default mode network (Yeo et al., 2011).

Fig. 2A shows the contrast between uncued semantic decisions and responses to letter strings (also uncued), while Fig. 3 shows the response to different cue contrasts (1 cue vs. 0 cues; 2 cues vs. 1 cue). The supplementary materials provide contrasts between semantic and letter string trials for each of the cue conditions separately (Figure S3). These maps show a similar semantic response across conditions, which resembles the contrast of 0 cues over letter strings.

The contrast between semantic decisions without cues and non-semantic trials revealed activation in brain areas previously associated with semantic cognition (in studies that largely did not employ cues; e.g. Binder et al., 2009; Noonan et al., 2013; Seghier et al., 2004; Bright et al., 2004; Gold et al., 2005; Chee et al., 2000; for reviews see Lambon Ralph et al., 2016; Jefferies, 2013; Hoffman et al., 2018), in left-hemisphere semantic areas such as inferior frontal gyrus and posterior temporal gyrus, as well as in medial temporal lobes, medial prefrontal and posterior cingulate cortex (Fig. 2A).

We then explored cueing effects by contrasting semantic decisions in the presence of different levels of constraint. The contrast of semantic decisions following 1 cue >0 cues identified clusters in task-positive regions overlapping with the MDN (Duncan, 2010), consistent with the cognitive demands of maintaining cues. We found recruitment of inferior and middle frontal gyrus (with the peak in inferior frontal sulcus), precentral gyrus, bilateral paracingulate gyrus and pre-supplementary motor area, temporo-occipital cortex and visual cortex. Interestingly, the effect of multiple cues compared with a single cue (2 ​> ​1) did not elicit stronger activation within these regions, even though the amount of information to be maintained was increased. Instead, this contrast elicited activation in regions overlapping with the DMN, including in bilateral angular gyrus/lateral occipital cortex, middle temporal gyrus, medial prefrontal cortex, posterior cingulate cortex, and left middle frontal gyrus. The thresholded maps for the two contrasts can be found in Fig. 3 (top panel). Parameter estimates for the three conditions over the implicit baseline were extracted in both the 1 ​> ​0 cue and 2 ​> ​1 cue regions (see Supplementary Figure S4). Overall, 1 ​> ​0 regions showed task-related activation (with more activation when cues had to be maintained in working memory, compared with the no cue condition) while 2 ​> ​1 regions exhibited task-related deactivation (with less deactivation when people made semantic decisions following 2 convergent cues compared with 0 or 1 cue).

We examined the overlap of the contrast maps with published maps of the MDN (Duncan, 2010) and DMN (Yeo et al., 2011, Fig. 3 - bottom). Consistent with the hypothesized role of DMN in semantic integration, 36.2% of the total voxels in the 2 ​> ​1 cue map overlapped with the DMN, while only 1% of voxels overlapped with MDN. For the 1 ​> ​0 cue map, the opposite pattern was observed, with 31.8% of total voxels overlapping with MDN and only 2.4% with DMN. We submitted the unthresholded z maps for the 2 ​> ​1 and 1 ​> ​0 cue contrasts to Neurosynth for cognitive decoding and produced word clouds using the top 10 terms positively associated with the maps (Fig. 3 – middle). The terms recovered for the 2 ​> ​1 and 1 ​> ​0 cue maps suggest the involvement of DMN and MDN respectively. The contrast of 2 ​> ​0 cues (Figure S3), shows activation in regions overlapping with 1 ​> ​0, such as left middle and inferior frontal gyrus, left middle temporal gyrus, but also in regions within the 2 ​> ​1 map, such as left angular gyrus. This pattern of activation suggests that both cue maintenance and cue integration might be visible in this map.

As the DMN is known to show anti-correlation with task-positive regions captured by the MDN (Blank et al., 2014; Davey et al., 2016; Fox et al., 2005), we also explored whether this would be the case for our contrast maps. In an independent sample of 86 participants, whole-brain connectivity maps for the 2 ​> ​1 and 1 ​> ​0 contrasts were generated using CONN (Whitfield-Gabrieli and Nieto-Castanon, 2012). Full methods are in the Supplementary Materials. The analysis revealed two functionally distinct and anti-correlated networks, comprising DMN for the 2 ​> ​1 cue contrast and MDN regions involved in domain-general executive control for the 1 ​> ​0 cue contrast (Figure S5).

##### 3.2.1.2 Cue model

To check whether the two distinct networks identified as relevant for conceptual cueing also showed different responses to load during the encoding of cue information, we constructed a second model to look at the cue presentation period. This was an exploratory analysis, since our main focus was on how cues modulate the neural basis of semantic decisions. Our paradigm was not designed to deconfound the order of the cues and the semantic decisions. Nevertheless, if the regions showing a stronger response to semantic decisions following 1 vs. 0 cues reflect the working memory demands of cue maintenance, we would expect to see load-dependent effects from cue encoding in these regions – i.e. stronger responses when more cues are presented.

First, we used a contrast of 2 cues +1 cue >0 cues across the whole brain to define the basic effect of cue presentation (see Fig. 2B). This elicited bilateral activation in occipital visual regions, extending into the posterior ventral stream in the left hemisphere. In addition, we found bilateral recruitment of the inferior frontal sulcus (IFS), within the multiple demand network, and the inferior frontal gyrus, in line with the idea of load demands of processing and maintaining cues (this interpretation is further explored in paragraph 3.2.2 ‘ROI analysis of cue load). Activation in the left hemisphere was also observed in AG.

The Supplementary Figure S2 shows other cue presentation contrasts. The contrast of 2 ​> ​0 cue presentation revealed activation in occipital cortex and in left-hemisphere control regions. Similar control regions were recruited by the contrast of 2 ​> ​1 cue presentation, although this map had less extensive activation overall. The contrast of 1 cue >0 cue presentation revealed activity in visual regions largely overlapping with 2 cues >0 cues, and a cluster in left angular gyrus. Finally, the contrast of 1 cue location >1 cue affect recruited visual regions in occipital cortex and bilateral paracingulate gyrus, while the reverse contrast did not yield significant results.

#### 3.2.2 ROI analysis of cue load

**Fig. 4:**
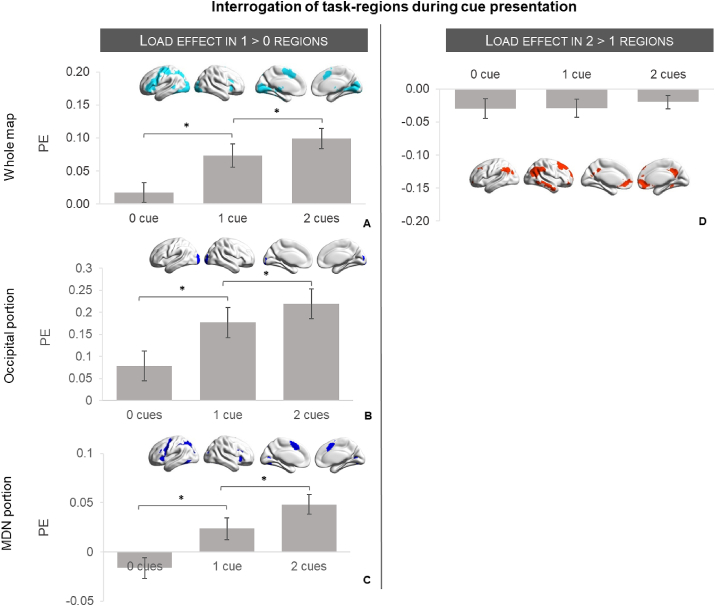
ROI analysis extracting the parameter estimates (PE) for the three levels of cue processing over the implicit baseline (cue model) in the 1 ​> ​0 and 2 ​> ​1 maps obtained in the semantic decision model. Three separate ROIs were conducted for the 1 ​> ​0 regions (left panel): whole map (A), voxels that fell within the occipital cortex (B) and voxels that fell in the MDN (C). While the effect of number of cues is present in the 1 ​> ​0 regions across the different masks used, no effect is observed in the integration regions (D) at the time-point of processing cue pictures. Bonferroni-corrected pairwise comparisons in the 1 ​> ​0 regions confirmed that PE for 0 cues were significantly lower than 1 cue, and PE for 1 cue were significantly lower than 2 cues (all p values ​< ​.025; p value corrected for 2 multiple comparisons).

To test possible accounts of the different patterns of activation observed in the decision-making phase (*semantic decision model*) for the contrasts of 2 ​> ​1 and 1 ​> ​0 cues, we conducted a post-hoc ROI analysis of the activation in these regions prior to the decision, when the cues were on the screen (*cue model*). The recruitment of cognitive control areas (i.e. inferior and middle frontal gyrus, inferior frontal sulcus, precentral gyrus, anterior cingulate gyrus, and pre-supplementary motor area, falling within the multiple demand network) for semantic decisions that followed the presence vs. absence of cues (1 ​> ​0 cues) suggests that these regions might be engaged in active maintenance of task-relevant information; in which case, cues might be processed in a load-dependent way during the cue period. To test this idea, the regions that responded to the contrasts of 1 ​> ​0 and 2 ​> ​1 cues during the semantic task (semantic decision model) were used to mask the BOLD response for cue presentation (cue model). We extracted and compared the parameter estimates for the three conditions against the implicit baseline: no cues (scrambled images), one cue (average of face emotion and location cue) and two cues (both face emotion and location image presented). If the semantic task activation observed for the 1 ​> ​0 contrast reflects a demand-relevant state associated with maintaining the cues, then the activation of these regions during cue presentation should increase as the number of cues is increased; i.e. 2 cues >1 cue, 1 cue >0 cues. This is because information about the cues is required to be maintained from their onset. In contrast, regions responding more to semantic decisions following multiple cues (2 ​> ​1 cues) might not be expected to show a load-dependent effect during the cue period. These regions responded more when multiple sources of information could be used to constrain semantic retrieval – and this form of information integration is unlikely to occur prior to the onset of the semantic decision (since the cues themselves were not easy to link in the absence of the probe concept – for example happy face and supermarket are consistent with a wide range of concepts and do not strongly prime jam). Consistent with these predictions, we found that activation in the 1 ​> ​0 cue regions increased in a linear fashion with a higher number of cues [F (1, 25) ​= ​48.39, p ​< ​.001, η^2^ ​= ​0.66] (Fig. 4A). However, there was no significant difference between cue conditions within regions responsive to the 2 ​> ​1 cue contrast [F (2, 50) ​= ​0.39, p ​= ​.682, η^2^ ​= ​0.02] (Fig. 4D).

The results of this ROI analysis show that regions responding more to semantic decisions following 1 ​> ​0 cues also respond in a load-dependent way during the encoding of cue information. However, this ROI map includes both cognitive control regions within MDN and visual cortex, making it difficult to separate the effects of increasing visual stimulation from cognitive load. To further characterize the effect, we divided the 1 ​> ​0 semantic decision map into regions that fell within the occipital cortex (Harvard-Oxford probabilistic map – 25%) and outside MDN regions (1997 voxels – Fig. 4B), and within MDN after masking out occipital regions (10658 voxels – Fig. 4C). The BOLD response showed a similar linear increase with the number of cues presented on the screen in visual cortex (F (1, 25) ​= ​54.96, p ​< ​.001, Ƞ^2^ ​= ​0.69) and in MDN (F (1, 25) ​= ​53.73, p ​< ​.001, Ƞ^2^ ​= ​0.68).

#### 3.2.3 Gradient analysis

**Fig. 5:**
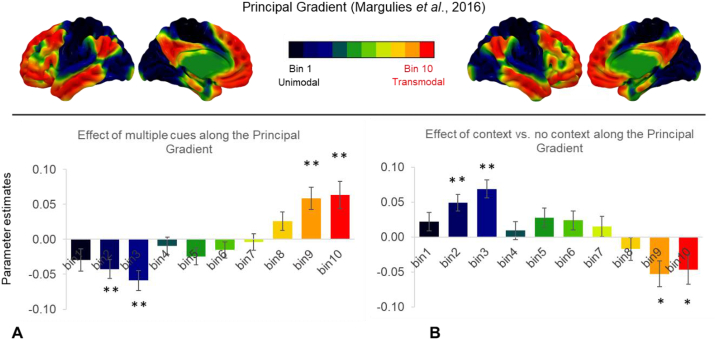
A. Semantic decisions in the presence of multiple cues (contrast of 2 ​> ​1 cues) maximally recruited regions at the heteromodal end of the principal gradient. B. The effect of context vs. no context (contrast of 1 ​> ​0 cues) showed an effect in the opposite direction, with maximal activation toward the sensory end of the gradient. ∗∗ Highlights portions of the gradient where the BOLD response is significantly different from 0 when the Bonferroni correction is applied (all p values ​≤ ​.005), while ∗ denotes p values ​< ​.05.

To further characterize the involvement of DMN regions in integrating information, we interrogated the response to semantic decisions along the Principal Gradient (Margulies et al., 2016). Unlike traditional univariate activation maps, which localize activation in certain regions, this gradient analysis examines how the effect of cueing unfolds along the entire cortical surface and measures the contribution of different portions of the gradient to the effects of interest. This analysis can highlight systematic functional change along the cortical surface, and explain why similar functional transitions are observed in multiple locations. The gradient map was divided into 10-percentile bins (see Methods section) and each bin was used as a mask in ROI analyses where we extracted mean parameter estimates for the contrasts of 2 cues vs. 1 cue and 1 cue vs. 0 cues within each bin (see Fig. 5). We then explored the effect of gradient bin on each univariate contrast using a two-way repeated measure ANOVA with *cue contrast* (2 levels: 2 cues vs.1 cue and 1 cue vs. 0 cues) and *gradient bin* (10 levels) as within-subject variables. This analysis revealed a significant interaction of cue contrast and gradient bin (F(2, 51) ​= ​28.33, p ​< ​.001, η^2^ ​= ​0.53), suggesting that the effect of gradient was different for 2 ​> ​1 and 1 ​> ​0 contrasts. Next, we performed two one-way repeated measure ANOVAs looking at the effect of *gradient bin* on each contrast separately. For 2 ​> ​1 cues, we found a significant linear effect for gradient bin (F(1, 25) ​= ​47.13, p ​< ​.001, η^2^ ​= ​0.65), as well as complex higher-order contrast effects (values reported in Table S5). The comparison of semantic decisions following 2 vs. 1 cues elicited maximal activity at the heteromodal end of the gradient, suggesting that DMN regions at this end of the principal gradient responded more strongly when multiple sources of information were integrated to support semantic cognition. For 1 ​> ​0 cues, we found the opposite pattern, with more activation at the unimodal end of the gradient for the single cue condition compared to when no cues were provided. Again, the effect of context vs. no-context along the principal gradient was complex, with linear (F(1, 25) ​= ​24.80, p ​< ​.001, η^2^ ​= ​0.50), as well as higher-order contrasts reaching significance. Full details of the statistical outcomes are reported in Supplementary Tables S4, S5, S6.

## 4 Discussion

Recent accounts of the default mode network (DMN) place this system at the top of a cortical hierarchy, maximally distant from unimodal sensory regions (Margulies et al., 2016) in both geodesic and connectivity space. The separation of heteromodal DMN regions from unimodal cortex may underpin our capacity to form conceptual representations that are not dominated by a particular type of feature but instead draw on multiple types of information – including affect or spatial location. To test this idea, we contrasted semantic decisions made following the presentation of multiple cues (depicting facial emotional expressions and locations), only one of these cues, or no cues. In this way, we manipulated the extent to which semantic retrieval occurred in a rich and meaningful context, in which multiple convergent features were available. Our results indicate that the cueing paradigm involved distinct mental processes that were supported by different networks. First, from the onset of the cues, information was maintained in working memory: MDN regions were activated for the contrast 1 cue >0 cues, and the response of these regions during cue presentation was load-dependent. These findings are in line with previous research showing that the multiple demand network supports the maintenance of goal-relevant information (Duncan, 2010; Woolgar et al., 2011). Secondly, DMN regions were activated by the contrast 2 cues >1 cue, consistent with a role of this network in convergent information integration. In line with our prediction that information integration occurs at the heteromodal end of the Principal Gradient, we found greater recruitment at this end when semantic decisions occurred in the presence of multiple cues. In contrast, activation was greater towards the unimodal end of the gradient (in regions overlapping with visual cortex) when semantic decisions were made in the presence vs. absence of cues. These novel findings provide important insights into the neural mechanisms supporting semantic integration and suggest a framework for understanding the location of these effects at the heteromodal end of the principal gradient.

According to “task-negative” accounts of the DMN, apparent semantic activation of this network occurs when an easy task is contrasted with a hard task (Humphreys et al., 2015; Humphreys et al., 2019; Humphreys and Lambon Ralph, 2014). This account is unlikely to provide an adequate explanation of our data since we found no behavioural differences between conditions (unlike other reports of cueing effects; Lanzoni et al., 2019; Noonan et al., 2010; Rodd et al., 2016, 2013). Our findings are instead consistent with a rich neuroimaging literature implicating ATL and AG in the formation of conceptual combinations. Integrating items (e.g. “jacket” and “plaid”) into coherent concepts (i.e. “plaid jacket”) modulates activity in AG regardless of the modality of presentation, while atrophy in this region results in impaired conceptual combinations (Price et al., 2015; see also Price et al., 2016). Similarly, magnetoencephalography (MEG) studies show increased activity in left ATL and AG for meaningful conceptual combinations (e.g. “red boat”) compared to the same words preceded by unpronounceable consonant strings (e.g. “xkq boat; Bemis and Pylkkänen, 2011, 2013; Pylkkänen, 2019), particularly when these combinations are more predictable or share more overlapping semantic features (Teige et al., 2019). Activation in the left superior ATL is also observed during semantic decisions following meaningful sentence cues, while IFG shows the opposite pattern (i.e. increased activation following irrelevant vs. relevant contexts), consistent with a role in semantic control (Hoffman et al., 2015). Moving beyond the language stimuli used in previous studies on conceptual combinations, here we show that semantic integration in DMN occurs for non-verbal material (i.e. pictures), in line with the heteromodal nature of these regions. Our findings uniquely add to this literature by showing that these effects of conceptual combination are maximal at the heteromodal end of the principal gradient, which situates DMN at the top of functional hierarchy (Margulies et al., 2016). Consequently, effects of information integration are seen not only in classic semantic regions such as AG and anterior middle temporal gyrus, but also in other DMN regions highlighted by our 2 ​> ​1 cues contrast (e.g., superior frontal gyrus; medial prefrontal and posterior cingulate cortex).

The role of DMN in semantic cognition appeared to be largely restricted to the impact of convergent cueing during semantic decision-making: in contrast, a distinct anti-correlated network overlapping with MDN was associated with the selective attention and working memory demands of encoding and maintaining individual cues. Moreover, the basic effect of making semantic decisions in the absence of cues, relative to the letter string trials, did not reveal activation in DMN regions. At first, this result may seem at odds with accounts of the DMN that attribute a crucial role in semantic cognition to this network. However, our semantic task was considerably more demanding than the letter string baseline: studies have shown that although DMN regions can respond to the contrast of semantic vs. non-semantic tasks, they typically do so when the semantic task is not more demanding than the comparison task (Binder et al., 2009; Humphreys et al., 2015). Moreover, activation in DMN regions is often associated with ‘automatic’ patterns of retrieval or conceptual combinations (Davey et al., 2016; Teige et al., 2019; Price et al., 2016, Bemis and Pylkkänen, 2011, 2013), while our task required participants to match an ambiguous words to a target word while discarding distractors and as such, it might involve more ‘controlled’ aspects of retrieval supported by regions such as left IFG which lie outside DMN.

In line with other studies, we found that DMN regions responding to cue integration (i.e. the 2 ​> ​1 cue contrast during semantic decisions) showed differential deactivation across conditions, relative to the implicit baseline, while MDN regions responding to cue maintenance (i.e., the 1 ​> ​0 cue contrast during semantic decisions) showed differential activation. The functional significance of task-related deactivation is a topic of considerable debate; while some authors have interpreted deactivation as suggesting that sites are irrelevant to ongoing cognition (e.g. Humphreys et al., 2015), another possibility is that deactivation might be functionally relevant, as it might allow DMN regions to integrate information more selectively from task-relevant networks (Krieger-Redwood et al., 2016). According to this “cognitive tuning” hypothesis, we might expect more deactivation of DMN regions when only a limited set of features are relevant to ongoing cognition (for example, in the 0 and 1 cue conditions, when emotion and location representations are not necessarily task-relevant). There are already studies demonstrating that DMN regions can increase their coupling to cognitive control areas when harder tasks are contrasted with easier tasks, even as they deactivate (Krieger-Redwood et al., 2016; Vatansever et al., 2015, 2017).

The effect of convergent cueing was not found within one specific semantic region, such as AG, but across multiple distributed nodes of DMN. We then turned to the Principal Gradient of intrinsic connectivity to provide a potential explanation for why cue integration effects were observed where they were across the cortex. The separation between DMN and unimodal systems, captured by the Principal Gradient, is thought to (i) allow heteromodal representations to emerge (cf. Hub and Spoke account) and (ii) support forms of cognition that require separation from the external environment, such as states that draw on heteromodal representations in memory. The latter observation is particularly important for explaining the similarity of our results with recent findings from our group (Murphy et al., 2018, 2019). Using a 1-back/0-back paradigm, Murphy et al., 2009 showed that decisions based on the immediately available perceptual input (0 back condition) elicited higher activity towards the unimodal end of the Principal Gradient, while decisions drawing on information from memory (1 back condition) maximally recruited the heteromodal end of the gradient (Murphy et al., 2019). Critically, DMN involvement in memory-guided cognition was maximised when the decisions involved meaningful objects that were not perceptually-identical, increasing reliance on conceptual knowledge, relative to simpler unidimensional decisions based on colour (Murphy et al., 2018). Building on these findings, the results of the current study suggest that this pattern of activation within DMN arises because heteromodal cortex at the top end of the gradient supports the integration of disparate and convergent sources of information; these regions are more involved when we match meaningful objects based on their identities extracted from a multitude of features, as opposed to single features. Nevertheless, Murphy et al. also showed that tasks based on memory recruit representations at the heteromodal end of the gradient, even when these tasks only probe a single feature and therefore arguably do not place strong demands on information integration: this pattern might arise because in the absence of perceptual inputs, heteromodal regions may play a key role in generating patterns of cognition needed for the task (i.e., visual imagery). Importantly, the regions at the top of the gradient responded similarly to memory-based decisions irrespective of whether these decisions concerned colour or shape; in this way, the function of these sites still reflects the heteromodal nature of DMN. In contrast, distinct unimodal sites responsive to colour and shape are expected to support these decisions when perceptual information is present. In summary, the principal gradient relating to the separation of heteromodal from unimodal processing can potentially explain both the increased response in heteromodal DMN when cognition involves multiple convergent features, and the common response in heteromodal DMN when cognition involves decisions about single features in the absence of perceptual input.

There are a number of limitations of this study. It does not fully establish the form of the relationship between the number of cues and DMN activation at retrieval, since we did not manipulate cueing parametrically. Activation in DMN regions may not increase linearly with the number of cues (0, 1, 2 cues). Instead, the contrast of 1 ​> ​0 cues elicits activation in MDN regions and towards the unimodal end of the principal gradient, suggesting that the presence vs. absence of context involves additional cue encoding and maintenance in working memory. A follow up study could use a parametric manipulation of the number of cues to better identify how responses in MDN and DMN scale with the number of cues. Moreover, in our experiment, integration unfolded over time, with semantic decisions occurring roughly 2 ​s after the presentation of the cue. A recent study by Branzi and others (2019) suggests that ventral AG supports the integration of meanings during time-extended narratives (see also Bonnici et al., 2016; Ramanan et al., 2017). Future research should establish whether semantic integration that emerges over time leads to a different pattern of activation along the principal gradient compared with the integration of simultaneously-presented information.

Furthermore, although our cueing paradigm allowed us to recover a set of regions within DMN recruited during semantic integration, it is unclear whether we would observe the same pattern of activation with other types of cues. Future studies could examine tasks that involve simple sensory features, for example, semantic decisions about concrete concepts such as dog following visual and auditory feature cues (e.g. image of tail and sound of dog barking) to establish if a similar integration effect occurs in DMN. The current experiment used complex stimuli depicting emotional affect and locations, which are known to be relevant to the DMN. The DMN is closely associated with the classic limbic network for emotional processing (e.g. Chanes and Barrett, 2016; Greicius et al., 2003; Raichle et al., 2001; Simpson et al., 2000). Moreover, the hippocampus, which has strong functional ties with the default mode network (Andrews-Hanna et al., 2010; Kernbach et al., 2018; Leech and Sharp, 2014; Raichle et al., 2001) is known to play a role in representing spatial locations (e.g. Bellmund et al., 2016; Burgess, 2002; Burgess et al., 2002; Robin et al., 2018). Our findings demonstrate that when semantic decisions are made in the context of both emotional and spatial information, as opposed to only one of these cue types, DMN ramps up its response in line with its hypothesized role in higher-order information integration. Contrary to previous literature showing the recruitment of the parahippocampal place area for spatial scenes (e.g. Epstein and Kanwisher, 1998) and the fusiform face area for faces (e.g. Kanwisher et al., 1997), our contrasts of 1 cue type over the other aligned only partially with previous evidence. The failure to reach statistical significance for the contrast of 1 cue affect >1 cue location could reflect a lack of statistical power, since much of the data acquisition was devoted to the semantic decisions. Moreover, the different size and aspect ratio of the images (with location images being wider and larger) may have influenced the results.

A final limitation of the study concerns the statistical model used to examine activation during cue presentation, which was used to test possible interpretations of the univariate results in the main model in a post-hoc fashion. As the experiment was not originally designed to look at the cue presentation, we did not include trials in which facial expressions and location cues were not followed by semantic decisions. Whenever a meaningful cue picture was presented, this was always followed by a semantic decision. The inclusion of trials where cues were followed by a blank screen would have facilitated the temporal separation of the cue and task events, allowing us to draw stronger conclusions from the cue model. In this way, future research could directly test the idea that integration requires a component of maintenance supported by the MDN, in addition to a combination of conceptual features within DMN.

## 5 Funding

The study was supported by a grant from the 10.13039/501100000364Stroke Association [R1425201] and a grant from the 10.13039/501100000781European Research Council [FLEXSEM – 771863] awarded to E.J.

## CRediT authorship contribution statement

**Lucilla Lanzoni:** Conceptualization, Methodology, Software, Validation, Formal analysis, Investigation, Data curation, Writing - original draft, Writing - review & editing, Visualization, Supervision, Project administration. **Daniela Ravasio:** Methodology, Investigation. **Hannah Thompson:** Methodology. **Deniz Vatansever:** Formal analysis, Writing - review & editing. **Daniel Margulies:** Conceptualization, Methodology. **Jonathan Smallwood:** Conceptualization, Resources, Writing - review & editing, Supervision. **Elizabeth Jefferies:** Conceptualization, Methodology, Resources, Writing - review & editing, Supervision, Funding acquisition.

## Declaration of competing interest

None.

## Footnotes

1 The images of spatial locations are not included in the collection due to potential copyright restrictions.
