# Supplementary material for: The role of default mode network in semantic cue integration
Source: Neuroimage. 2020 Oct 1;219:117019. doi: 10.1016/j.neuroimage.2020.117019 (PMC7443705; doi:10.1016/j.neuroimage.2020.117019)
Supplement: Multimedia component 1 [file mmc1.docx]

**Supplementary material**

Group behavioural performance


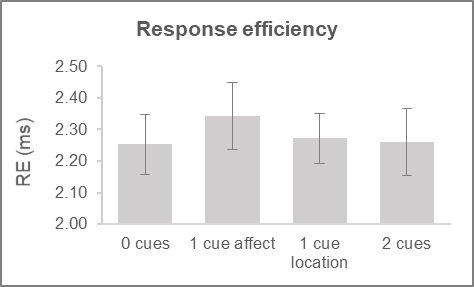

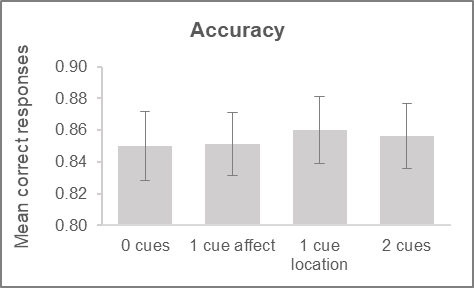

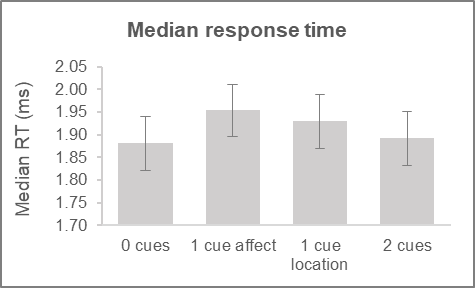

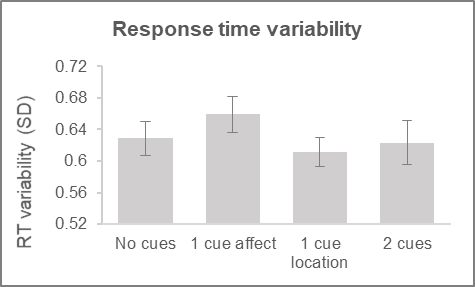


Figure S1. Accuracy (mean correct responses), median RT for correct trials (milliseconds), response efficiency scores (median RT/mean correct responses), and RT variability (mean standard deviation per participant per condition) do not differ significantly across conditions. Error bars show standard error of the mean (SEM).

|  | **Summary statistics** | | | |
| --- | --- | --- | --- | --- |
|  | **Accuracy** | **Median RT** | **Response efficiency** | **RT variability** |
| 0 cues | 0.85 (0.11) | 1.88 (0.30) | 2.25 (0.49) | 0.63 (0.11) |
| 1 cue affect | 0.85 (0.10) | 1.95 (0.29) | 2.34 (0.54) | 0.66 (0.12) |
| 1 cue location | 0.86 (0.11) | 1.93 (0.31) | 2.27 (0.41) | 0.61 (0.10) |
| 2 cues | 0.86 (0.10) | 1.89 (0.30) | 2.26 (0.54) | 0.62 (0.14) |

Table S1. Descriptive statistics for the cueing task. Mean and (standard deviation) values are provided.

Supplementary behavioural analyses

|  | **1- way repeated measures ANOVAs on cue condition** | | | |
| --- | --- | --- | --- | --- |
|  | **Accuracy** | **Median RT** | **Response efficiency** | **RT variability** |
| F | 0.14 | 0.95 | 0.62 | 1.26 |
| df | 3, 75 | 3, 75 | 3, 75 | 3, 75 |
| p | 0.939 | 0.420 | 0.605 | 0.296 |
| partial η2 | 0.01 | 0.04 | 0.02 | 0.05 |

Table S2. Behavioural performance did not differ significantly across cue conditions, as revealed by 1-way ANOVAs on accuracy, median response time, response efficiency, and response time variability.

Univariate contrasts of activation during cue presentation

Below we report the group-level statistical maps (z > 3.1) for the cue model. In this model we looked at changes in the BOLD response in response to the presentation of the visual cues. Semantic decisions (which happen subsequently to the presentation of cues) were modelled separately; the statistical maps that survived the threshold of z > 3.1 can be seen in the body of the manuscript (Figure 2B and 4).


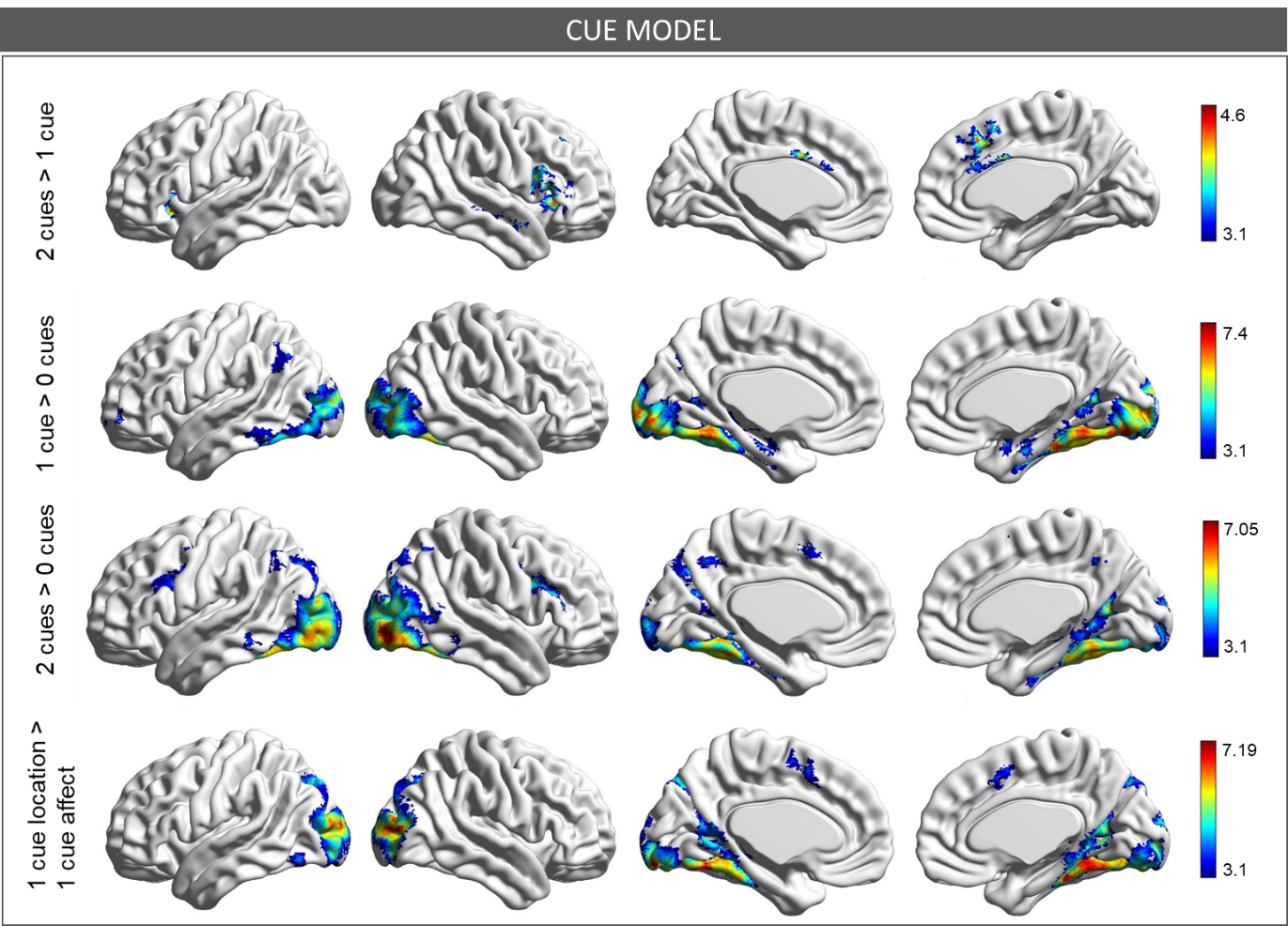


Figure S2. Univariate results for the cue model (i.e. when the cues were presented, prior to the semantic decision). From top to bottom: 2 cues > 1 cue (processing of 2 cues > 1 cue [average of affect and location]), 1 cue > 0 cues (processing of 1 cue [average of affect and location] > 0 cues [scrambled images]); 2 cues > 0 cues; 1 cue location > 1 cue affect. The reverse contrast (1 cue affect > 1 cue location) yielded no clusters. Coordinates of cluster peaks for these comparisons are reported in Table S3.

Basic effect of semantic decisions

In the main manuscript we defined the semantic regions recruited during the task using a contrast of 0 cues > letter strings (Figure 2A). Below we report the contrasts of each of the other task conditions against the presentation of letter strings (i.e. non semantic task).


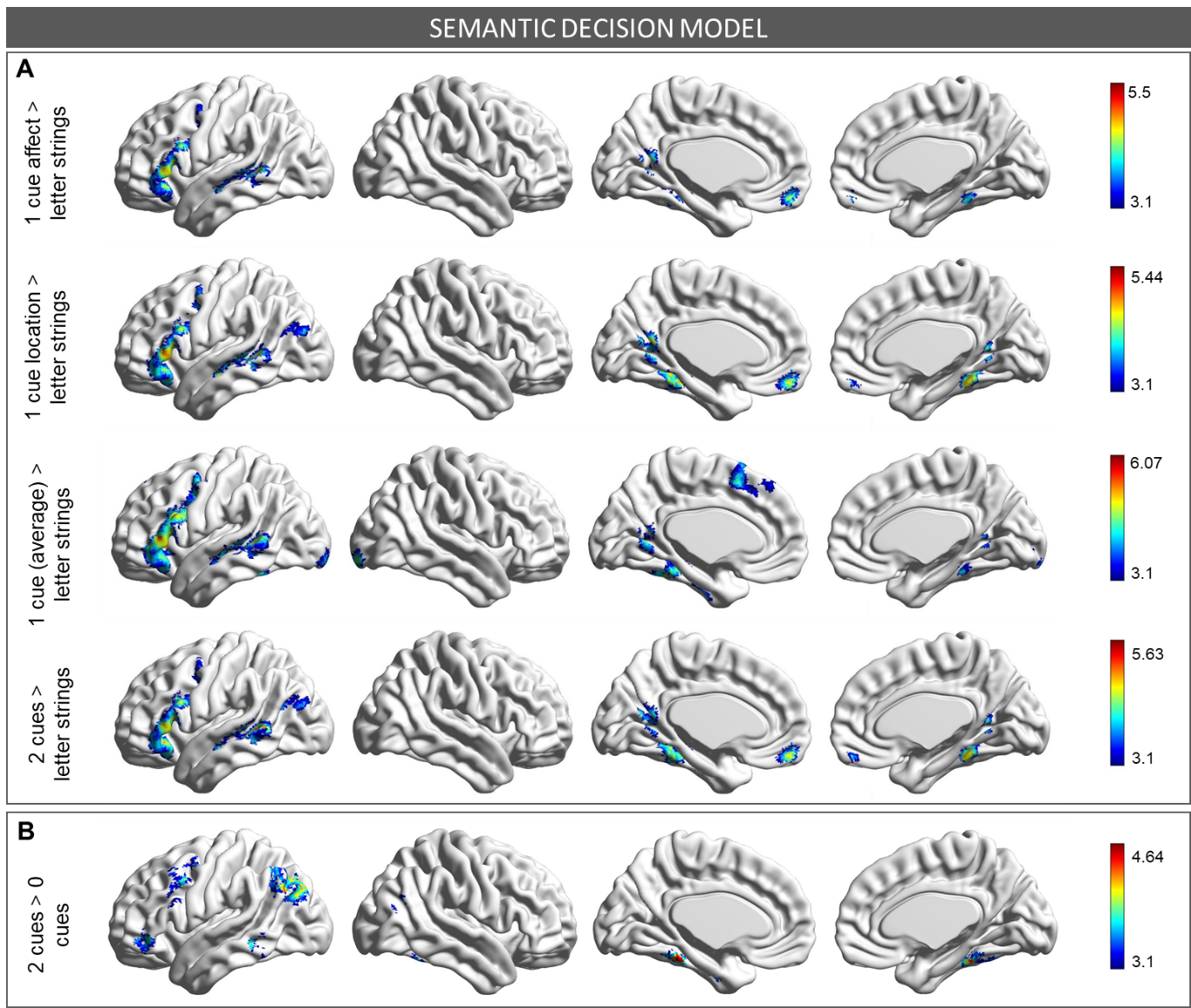


Figure S3. A. Basic effect of semantic decisions as estimated by contrasts of the task conditions > letter strings (i.e. non-semantic task). These univariate contrasts for the semantic decision model (i.e. when participants were making decisions following the presentation of 0, 1, 2 cues) reveal a similar pattern of activation. B. Semantic decisions following 2 cues vs. 0 cues.


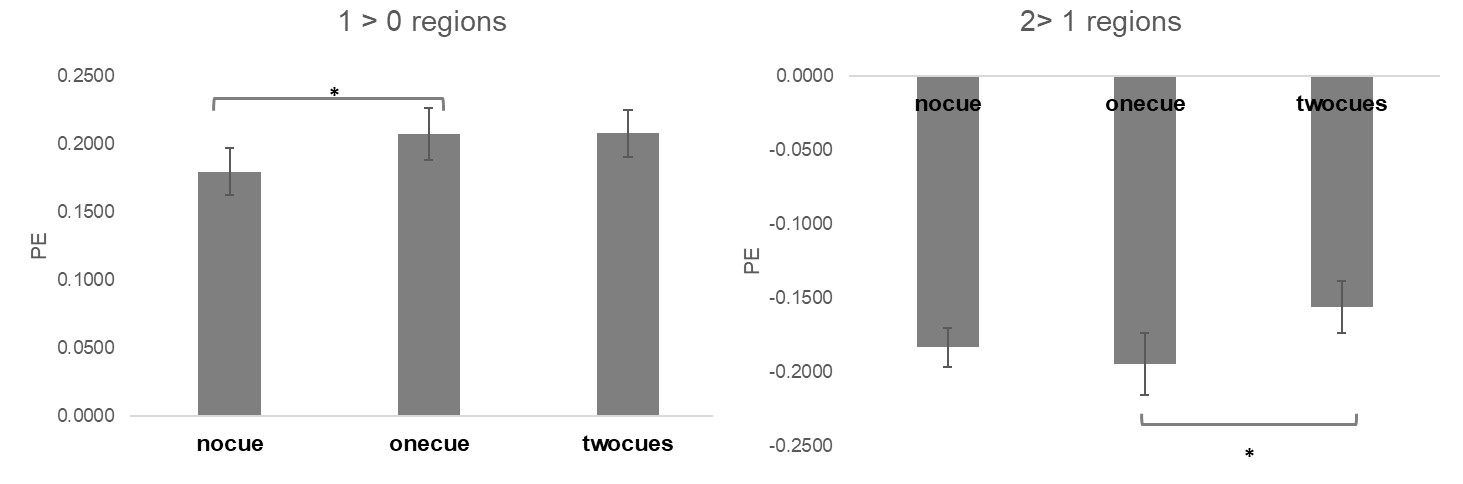


Figure S4. ROI analysis extracting the parameter estimates (PE) for the three conditions over the implicit baseline at the time of making semantic decisions (semantic decision model) in the 1 > 0 and 2 > 1 maps. We ran two repeated measures ANOVAs that found that the recruitment is different across cueing conditions in both 1 > 0 [F (2,50) = 3.36, p = .043, η^2^ = .12] and 2 > 1 [F (2,50) = 3.47, p = .039, η^2^ = .12] regions. Bonferroni-corrected pairwise comparisons revealed reduced activation for 0 cue condition compared to 1 cue in 1 > 0 regions [t(25) = -2.59, p = .016], and reduced de-activation in 2 cues compared to 1 cue in 2 > 1 regions [t(25) = -3.03, p = .006].

Peak co-ordinates for clusters identified by the cue model and the semantic decision model

| Contrast | Region | Voxels | Z-score | MNI coordinates (x, y, z) | | |
| --- | --- | --- | --- | --- | --- | --- |
| *Cue model* | | | | | | |
| 2 + 1 > 0 | R. Lingual gyrus, occipital fusiform gyrus, occipital pole | 26628 | 7.49 | 8 | -82 | -12 |
|  | L. Middle frontal gyrus, superior frontal gyrus | 769 | 4.51 | -36 | 22 | 54 |
|  | L. Frontal pole | 766 | 4.99 | -42 | 54 | -4 |
|  | L. temporal pole, inferior temporal gyrus (anterior), temporal fusiform gyrus (anterior) | 130 | 4.29 | -42 | 4 | -42 |
| 2 > 0 | R. Lateral occipital cortex (inferior), occipital pole | 21099 | 7.05 | 42 | -86 | -8 |
|  | R. Precentral gyrus, inferior frontal gyrus, middle frontal gyrus | 866 | 5.11 | 38 | 10 | 28 |
|  | L. Inferior frontal gyrus (pars opercularis), precentral gyrus, middle frontal gyrus | 850 | 4.63 | -40 | 16 | 22 |
|  | Bilateral precuneus | 189 | 4.11 | 0 | -56 | 46 |
|  | L. Supplementary motor cortex, paracingulate gyrus, superior frontal gyrus | 177 | 4.29 | -4 | 6 | 54 |
| 2 > 1 | R. Frontal operculum cortex, inferior frontal gyrus (pars opercularis), inferior frontal gyrus (pars triangularis) | 940 | 4.48 | 46 | 18 | 6 |
|  | R. Paracingulate gyrus, superior frontal gyrus, cingulate gyrus (anterior) | 585 | 4.38 | 6 | 24 | 44 |
|  | R. Middle frontal gyrus, superior frontal gyrus, frontal pole | 267 | 4.55 | 28 | 32 | 44 |
|  | R. Superior temporal gyrus (anterior), middle temporal gyrus (anterior), superior temporal gyrus (posterior), middle temporal gyrus (posterior) | 199 | 4.35 | 54 | -6 | -16 |
|  | L. Insular cortex | 152 | 4.51 | -28 | 20 | -6 |
| 1 > 0 | R. Occipital fusiform gyrus, lingual gyrus | 21130 | 7.38 | 24 | -72 | -14 |
|  | L. Lateral occipital cortex (superior), angular gyrus | 229 | 4 | -50 | -66 | 40 |
|  | L. Frontal pole | 229 | 4.51 | -40 | 58 | 2 |
|  | R. Cerebellum | 198 | 4.24 | 28 | -74 | -44 |
| 1 location > 1 affect | R. Occipital pole, lateral occipital cortex | 17891 | 7.19 | 36 | -88 | 8 |
|  | Bilateral paracingulate gyrus, supplementary motor cortex | 411 | 4.31 | 0 | 10 | 50 |
| *Semantic decision model* | | | | | | |
| 0 > letter strings | L. Inferior frontal gyrus (pars triangularis), frontal pole, middle frontal gyrus | 1672 | 5.92 | -54 | 32 | 8 |
|  | L. Superior temporal gyrus (posterior), middle temporal gyrus (posterior), supramarginal gyrus (posterior) | 953 | 5.52 | -56 | -42 | 4 |
|  | R. Cerebellum | 644 | 5.04 | 10 | -82 | -36 |
|  | L. Temporal fusiform cortex (posterior), parahippocampal gyrus (posterior), inferior temporal gyrus (posterior) | 400 | 4.51 | -38 | -30 | -18 |
|  | L. Cerebellum | 280 | 4.62 | -8 | -60 | 14 |
|  | L. Frontal medial cortex, frontal pole | 256 | 4.66 | -4 | 52 | -16 |
|  | R. Temporal occipital fusiform, Lingual gyrus, parahippocampal gyrus (posterior) | 220 | 4.91 | 22 | -42 | -16 |
|  | L. Precentral gyrus, middle frontal gyrus | 153 | 4.7 | -38 | 0 | 46 |
| 1 affect > letter strings | L. Inferior frontal gyrus (pars triangularis), frontal pole, middle frontal gyrus | 1684 | 4.91 | -56 | 32 | 6 |
|  | L. Middle temporal gyrus (temporo-occipital part), middle temporal gyrus (posterior) | 926 | 5.5 | -56 | -44 | 4 |
|  | R. Cerebellum | 571 | 4.81 | 10 | -82 | -36 |
|  | L. Temporal fusiform (posterior), parahippocampal gyrus (posterior), inferior temporal gyrus (posterior) | 261 | 4.2 | -38 | -30 | -18 |
|  | L. Precuneus, intracalcarine cortex, supracalcarine cortex, cingulate gyrus (posterior) | 258 | 4.65 | -8 | -60 | 14 |
|  | L. Medial frontal cortex, frontal pole | 186 | 4.6 | -2 | 52 | -16 |
|  | R. Temporal fusiform (posterior), parahippocampal gyrus (posterior), lingual gyrus | 138 | 4.19 | 24 | -38 | -18 |
|  | L. Precentral gyrus, middle frontal gyrus | 125 | 4.11 | -38 | 0 | 44 |
| 1 location > letter strings | L. Inferior frontal gyrus (pars triangularis), frontal pole, middle frontal gyrus | 2000 | 5.44 | -56 | 32 | 8 |
|  | L. Middle temporal gyrus (temporo-occipital part), middle temporal gyrus (posterior) | 1107 | 5.04 | -58 | -44 | 4 |
|  | R. Cerebellum | 910 | 5.12 | 12 | -78 | -30 |
|  | L. Temporal fusiform (posterior), parahippocampal gyrus (posterior), inferior temporal gyrus (posterior) | 773 | 5.06 | -38 | -30 | -18 |
|  | L. Precuneus, intracalcarine cortex, lingual gyrus, supracalcarine cortex, cingulate gyrus (posterior) | 723 | 4.93 | -8 | -60 | 10 |
|  | R. Lingual gyrus, occipital fusiform gyrus, parahippocampal gyrus (posterior), temporal fusiform (posterior) | 375 | 5.4 | 20 | -40 | -16 |
|  | L. Medial frontal cortex, frontal pole | 230 | 4.71 | -2 | 52 | -16 |
|  | L. Precentral gyrus, middle frontal gyrus | 205 | 4.61 | -38 | 0 | 44 |
|  | L. Lateral occipital cortex (superior) | 176 | 3.97 | -44 | -84 | 26 |
| 1 > letter strings | L. Inferior frontal gyrus (pars triangularis), frontal pole, middle frontal gyrus | 3459 | 5.65 | -54 | 30 | 8 |
|  | L. Middle temporal gyrus (temporo-occipital part), middle temporal gyrus (posterior) | 2493 | 5.41 | -56 | -44 | 4 |
|  | R. Cerebellum | 1165 | 6.07 | 12 | -78 | -28 |
|  | L. Precuneus, lingual gyrus, intracalcarine cortex, cingulate gyrus (posterior), supracalcarine cortex | 371 | 4.73 | -6 | -58 | 8 |
|  | L. Paracingulate gyrus, superior frontal gyrus, juxtapositional lobule | 337 | 4.56 | -6 | 14 | 52 |
|  | R. Occipital pole | 275 | 4.89 | 18 | -100 | -12 |
|  | L. Occipital pole | 235 | 4.91 | -34 | -98 | -14 |
|  | R. Precuneus, lingual gyrus, intracalcarine cortex, cingulate gyrus (posterior), supracalcarine cortex | 127 | 4.54 | 14 | -56 | 6 |
|  | R. temporal fusiform (posterior), parahippocampal gyrus (posterior), lingual gyrus | 118 | 4.37 | 28 | -36 | -20 |
| 2 > letter strings | L. Inferior frontal gyrus (pars triangularis), frontal pole, middle frontal gyrus | 2239 | 5.51 | -56 | 32 | 8 |
|  | L. Middle temporal gyrus (temporo-occipital), middle temporal gyrus (posterior), supra-marginal gyrus (posterior), , superior temporal gyrus (posterior) | 1175 | 5.63 | -48 | -44 | -2 |
|  | R. Cerebellum | 794 | 4.79 | 10 | -82 | -34 |
|  | L. Temporal fusiform (posterior), temporal occipital fusiform, parahippocampal gyrus (posterior), lingual gyrus | 695 | 4.95 | -26 | -42 | -20 |
|  | L. Precuneus, lingual gyrus, intracalcarine cortex, cingulate gyrus (posterior), supracalcarine cortex | 458 | 4.77 | -8 | -58 | 10 |
|  | R. Temporal occipital fusiform, Lingual gyrus, temporal fusiform (posterior), parahippocampal gyrus (posterior) | 367 | 5.6 | 22 | -40 | -16 |
|  | L. Angular gyrus, lateral occipital cortex (superior), lateral occipital cortex (inferior) | 265 | 4.4 | -40 | -60 | 18 |
|  | L. Frontal medial cortex, frontal pole | 237 | 4.82 | -2 | 52 | -16 |
|  | R. Precuneus, Intracalcarine cortex, cingulate gyrus (posterior), lingual gyrus, supracalcarine cortex | 201 | 4.67 | 16 | -54 | 8 |
| 2 > 1 | R. Lateral occipital cortex (superior) | 5247 | 5.94 | 52 | -70 | 30 |
|  | R. Frontal pole, paracingulate gyrus, frontal medial cortex | 4395 | 5.57 | 4 | 56 | 0 |
|  | L. lateral occipital cortex (superior), angular gyrus, supramarginal gyrus (posterior) | 1639 | 5.27 | -50 | -62 | 42 |
|  | R. Precuneus, cingulate gyrus (posterior) | 1521 | 5.17 | 8 | -56 | 26 |
|  | L. Cerebellum | 745 | 4.77 | -26 | -78 | -36 |
|  | L. Middle frontal gyrus | 304 | 4.46 | -36 | 26 | 42 |
|  | R. Frontal pole | 191 | 4.35 | 40 | 48 | -10 |
|  | L. Frontal pole | 172 | 4.33 | -30 | 62 | -2 |
|  | R. Temporo-occipital fusiform, lingual gyrus, parahippocampal gyrus | 143 | 4.72 | 24 | -42 | -16 |
| 1 > 0 | R. Cerebellum | 30503 | 6.81 | 4 | -74 | -28 |
|  | L. Supplementary motor cortex, paracingulate gyrus, superior frontal gyrus, cingulate gyrus (anterior) | 2167 | 6.57 | -4 | 8 | 52 |
|  | R. Inferior frontal gyrus (pars opercularis), middle frontal gyrus, inferior frontal gyrus (pars triangularis), precentral gyrus | 714 | 5.59 | 42 | 22 | 20 |
|  | L. Middle temporal gyrus (temporo-occipital), supra-marginal gyrus (posterior), middle temporal gyrus (posterior), superior temporal gyrus | 254 | 4.85 | -56 | -46 | 4 |
| 2 > 0 | L. Lateral occipital cortex (superior), angular gyrus | 1431 | 4.55 | -46 | -66 | 32 |
|  | R. Lateral occipital cortex (inferior), occipital fusiform gyrus | 533 | 4.55 | 38 | -74 | -24 |
|  | L. Middle frontal gyrus, precentral gyrus, inferior frontal gyrus (pars opercularis) | 472 | 4.04 | -46 | 12 | 40 |
|  | L. Temporal fusiform cortex (posterior), temporal occipital fusiform cortex, parahippocampal gyrus (posterior) | 441 | 4.64 | -26 | -40 | -20 |
|  | L. Occipital fusiform gyrus | 283 | 4.55 | -46 | -72 | -26 |
|  | L. Frontal pole, frontal orbital cortex, inferior frontal gyrus (pars triangularis) | 153 | 3.94 | -52 | 40 | -8 |
|  | L. Middle temporal gyrus (posterior), middle temporal gyrus (temporooccipital) | 128 | 3.97 | -60 | -40 | -8 |
|  | R. Lateral occipital cortex (superior), angular gyrus | 125 | 3.94 | 52 | -64 | 24 |

Table S3. Coordinates of cluster peaks for the main contrasts of interest. From top to bottom: cue model – 2 + 1 > 0 (processing of cues > scrambles images), 2 > 0 (processing of 2 cues [affect and location] > 0 cues [scrambled images]), 2 > 1 (processing of 2 cues [affect and location] > 1 cue [average of affect and location]), 1 > 0 (processing of 1 cue [average of affect and location] > 0 cues [scrambled images]), 1 affect > 1 location; semantic decision model - 0 cues > letter strings (semantic decisions in the absence of a semantic cue > non semantic decisions in the absence of cues), 1 affect > letter strings, 1 location > letter strings, 1 > letter strings (semantic decision following 1 cue [average of affect and location] > non semantic decisions in the absence of cues), 2 > letter strings, 2 > 1 (semantic decisions following multiple cues > semantic decisions following 1 cue), 1 > 0 (semantic decisions following 1 semantic cue > semantic decisions in the absence of a semantic cue). The location of the peaks is labelled according to the Harvard-Oxford Structural Cortical Atlas tool available in FSL. Caption: R = right hemisphere, L = left hemisphere, cluster corrected at z > 3.1.

Supplementary analyses examining activation for the semantic task along the Principal Gradient

|  | **2 (cue contrast: 2 vs. 1, 1 vs. 0) x 10 (gradient bin: bin1 - bin10) ANOVA** | | | | | |  |
| --- | --- | --- | --- | --- | --- | --- | --- |
|  | **Test of within-subjects effect** | | | **Test of within-subjects contrasts** | | |  |
|  | Cue contrast | Gradient bin | Cue contrast x gradient bin | Cue contrast | Gradient bin | Cue contrast x gradient bin |  |
| F | 0.33 | 1.82 | 28.33 | 0.33 | 1.53 | 37.27 | Linear |
| p | .571 | .164 | <.001* | .571 | .227 | <.001* |  |
| partial η^2^ | 0.01 | 0.07 | 0.53 | 0.01 | 0.06 | 0.60 |  |
| F |  |  |  |  | 0.06 | 12.37 | Quadratic |
| p |  |  |  |  | .815 | .002* |  |
| partial η^2^ |  |  |  |  | 0.00 | 0.33 |  |
| F |  |  |  |  | 2.26 | 6.47 | Cubic |
| p |  |  |  |  | .145 | .018* |  |
| partial η^2^ |  |  |  |  | 0.08 | 0.21 |  |
| F |  |  |  |  | 0.79 | 0.28 | Order 4 |
| p |  |  |  |  | .382 | .601 |  |
| partial η^2^ |  |  |  |  | 0.03 | 0.01 |  |
| F |  |  |  |  | 20.44 | 111.60 | Order 5 |
| p |  |  |  |  | <.001* | <.001* |  |
| partial η^2^ |  |  |  |  | 0.45 | 0.82 |  |
| F |  |  |  |  | 1.06 | 9.51 | Order 6 |
| p |  |  |  |  | .312 | .005* |  |
| partial η^2^ |  |  |  |  | 0.04 | 0.28 |  |
| F |  |  |  |  | 5.07 | 38.85 | Order 7 |
| p |  |  |  |  | .033* | <.001* |  |
| partial η^2^ |  |  |  |  | 0.17 | 0.61 |  |
| F |  |  |  |  | 2.50 | 64.54 | Order 8 |
| p |  |  |  |  | .127 | <.001* |  |
| partial η^2^ |  |  |  |  | 0.09 | 0.72 |  |
| F |  |  |  |  | 1.07 | 66.73 | Order 9 |
| p |  |  |  |  | .311 | <.001* |  |
| partial η^2^ |  |  |  |  | 0.04 | 0.73 |  |

Table S4. Values for the 2-way repeated measure ANOVA on cue contrast (2 levels: 2 cues > 1 cue; 1 cue > 0 cues) and gradient bin (10 levels: bin1 – bin10). Degrees of freedom for the Test of Within-subjects Effects: cue condition [1, 25]; gradient bin [2.37, 59.16]; cue contrast x gradient bin [2.04, 51.01]. Degrees of freedom for the Test of Within-subjects Contrasts: cue contrast, gradient bin, cue contrast x gradient bin [1, 25]. Significant results and interactions are marked with *. A Greenhouse-Geisser correction was applied where the assumption of sphericity was not met.

| **1 way RM ANOVA on 2 cues > 1 cue along the gradient** | | | |
| --- | --- | --- | --- |
|  | **Test of within-subjects effect** | **Test of within-subjects contrasts** |  |
|  | Gradient bin | Gradient bin |  |
| F | 31.50 | 47.13 | Linear |
| p | <.001* | <.001* |  |
| partial η^2^ | 0.56 | 0.65 |  |
| F |  | 11.38 | Quadratic |
| p |  | .002* |  |
| partial η^2^ |  | 0.31 |  |
| F |  | 3.03 | Cubic |
| p |  | .094 |  |
| partial η^2^ |  | 0.11 |  |
| F |  | 0.06 | Order 4 |
| p |  | .813 |  |
| partial η^2^ |  | 0.00 |  |
| F |  | 70.22 | Order 5 |
| p |  | <.001* |  |
| partial η^2^ |  | 0.74 |  |
| F |  | 7.66 | Order 6 |
| p |  | .010* |  |
| partial η^2^ |  | 0.23 |  |
| F |  | 31.30 | Order 7 |
| p |  | <.001* |  |
| partial η^2^ |  | 0.56 |  |
| F |  | 48.60 | Order 8 |
| p |  | <.001* |  |
| partial η^2^ |  | 0.66 |  |
| F |  | 50.58 | Order 9 |
| p |  | <.001* |  |
| partial η^2^ |  | 0.67 |  |

Table S5. Values for the 1-way repeated measure ANOVA on the parameter estimates for the univariate contrast of 2 cues > 1 cue extracted along the gradient (10 levels: bin1 – bin10). Degrees of freedom for the Test of Within-subjects Effects: 2.13, 53.30. Degrees of freedom for the Test of Within-subjects Contrasts: 1, 25. Significant results and interactions are marked with *. A Greenhouse-Geisser correction was applied where the assumption of sphericity was not met.

| **1 way RM ANOVA on 1 cue > 0 cues along the gradient** | | | |
| --- | --- | --- | --- |
|  | **Test of within-subjects effect** | **Test of within-subjects contrasts** |  |
|  | Gradient bin | Gradient bin |  |
| F | 21.37 | 24.80 | Linear |
| p | <.001* | <.001* |  |
| partial η^2^ | 0.46 | 0.50 |  |
| F |  | 11.48 | Quadratic |
| p |  | .002* |  |
| partial η^2^ |  | 0.31 |  |
| F |  | 7.44 | Cubic |
| p |  | .011* |  |
| partial η^2^ |  | 0.23 |  |
| F |  | 0.52 | Order 4 |
| p |  | .478 |  |
| partial η^2^ |  | 0.02 |  |
| F |  | 116.31 | Order 5 |
| p |  | <.001* |  |
| partial η^2^ |  | 0.82 |  |
| F |  | 8.61 | Order 6 |
| p |  | .007* |  |
| partial η^2^ |  | 0.26 |  |
| F |  | 40.27 | Order 7 |
| p |  | <.001* |  |
| partial η^2^ |  | 0.62 |  |
| F |  | 62.44 | Order 8 |
| p |  | <.001* |  |
| partial η^2^ |  | 0.71 |  |
| F |  | 64.32 | Order 9 |
| p |  | <.001* |  |
| partial η^2^ |  | 0.72 |  |

Table S6. Values for the 1-way repeated measure ANOVA on the parameter estimates for the univariate contrast of 1 cue > 0 cues extracted along the gradient (10 levels: bin1 – bin10). Degrees of freedom for the Test of Within-subjects Effects: 2.05, 51.22. Degrees of freedom for the Test of Within-subjects Contrasts: 1, 25. Significant results and interactions are marked with *. A Greenhouse-Geisser correction was applied where the assumption of sphericity was not met.

**Analysis of intrinsic connectivity**

As there is evidence that DMN is anti-correlated with task-positive regions captured by MDN (Blank et al., 2014; Davey et al., 2016; Fox et al., 2005), we predicted that our contrast maps of 1 > 0 and 2>1 should fall within regions with distinct patterns of intrinsic connectivity at rest, given their spatial similarity with the MDN and with the DMN, respectively.

***Materials and Methods***

*Participants*

Whole-brain intrinsic connectivity maps for the two contrasts (1 >0 and 2>1) were produced using a sample of 86 participants recruited as part of a different study. The study was approved by the Ethics Committees of the York Neuroimaging Centre and the Department of Psychology, University of York. Volunteers provided written consent and were debriefed after the experiment.

*MRI data acquisition*

Structural and functional MRI data were acquired using a 3T GE HDx Excite MRI scanner at the York Neuroimaging Centre, University of York. Structural MRI acquisition was based on the same protocol used for the main sample of this experiment (see Materials and Methods*– fMRI acquisition*). Resting-state fMRI data was recorded from the whole brain using single-shot 2D gradient-echo-planar imaging (TR=3s, TE=minimum full, flip angle=90°, matrix size=64x64, 60 slices, voxel size=3x3x3mm3, 180 volumes). Participants were asked to passively view a fixation cross and not to think of anything in particular for the duration of the resting-state scan (9 minutes). A T1 weighted FLAIR scan with the same orientation as the functional scans was collected to improve co-registration between subject-specific structural and functional scans (TR=2560ms, TE=minimum full, matrix size=64x64, voxel size=3x3x3mm3).

*Pre-processing*

The pre-processing of resting state data was performed using the CONN functional connectivity toolbox V.18a (<http://www.nitrc.org/projects/conn>; Whitfield-Gabrieli & Nieto-Castanon, 2012). The following steps were performed on the functional volumes: (1) slice-time (bottom-up, interleaved) and motion-correction, (2) skull-stripping and co-registration to the high-resolution structural image, (3) spatial normalisation to Montreal Neurological Institute (MNI) space using the unified-segmentation algorithm, (4) smoothing with a 6mm FWHM Gaussian kernel, and (5) band-passed filtering (0.008-0.09Hz) to reduce low-frequency drift and noise effects. Nuisance regressors in the pre-processing pipeline included: (i) motion (12 parameters: the six translation and rotation parameters and their temporal derivatives), (ii) scrubbing (outliers were identified through the artefact detection algorithm included in CONN based on scan-by-scan change in global signal above z=3, subject motion threshold above 5mm, differential motion and composite motion exceeding 95% percentile in the normative sample), (iii) CompCor components (the first 5) attributable to the signal from white matter and CSF (Behzadi et al., 2007), and (iv) a linear detrending term, eliminating the need for global signal normalisation (Chai et al., 2012; Murphy et al., 2009).


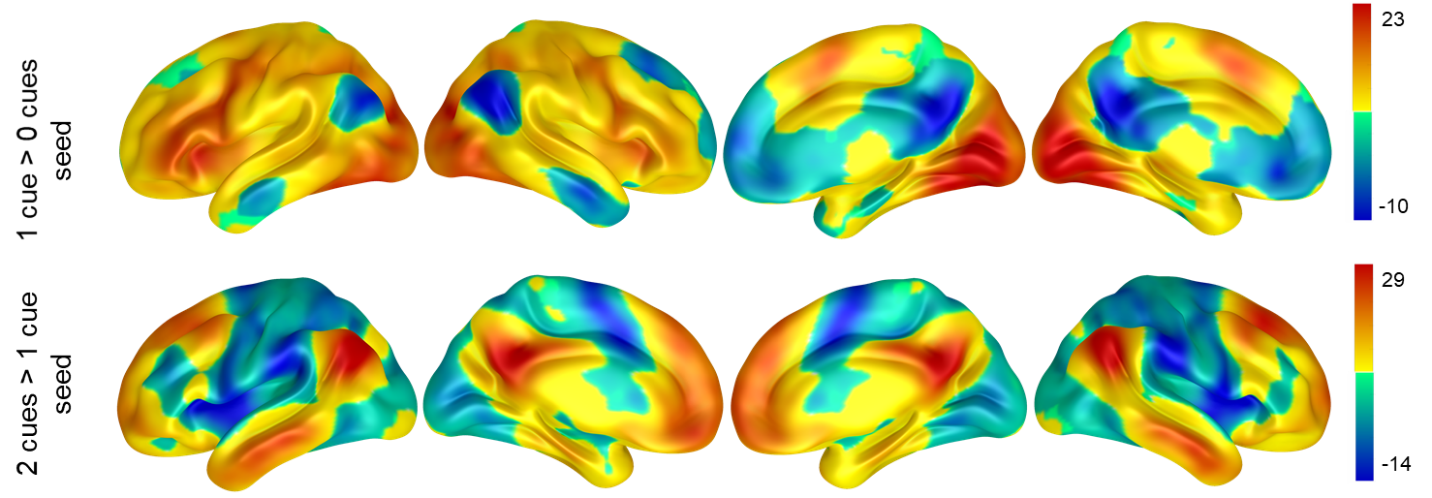


Figure S5. Intrinsic connectivity maps obtained in a separate sample of 86 participants using the thresholded (z > 3.1) contrast maps of 2 cues > 1cue and 1 cue > 0 as seeds in a resting state analysis. These reveal two functionally distinct and anti-correlated networks, comprising multiple demand regions for 1>0 and default mode regions for 2>1.

**BIBLIOGRAPHY**

Behzadi, Y., Restom, K., Liau, J., & Liu, T. T. (2007). A component based noise correction method (CompCor) for BOLD and perfusion based fMRI. *Neuroimage*, *37*(1), 90-101. <https://doi.org/10.1016/j.neuroimage.2007.04.042>

Blank, I., Kanwisher, N., & Fedorenko, E. (2014). A functional dissociation between language and multiple-demand systems revealed in patterns of BOLD signal fluctuations. *Journal of neurophysiology*, *112*(5), 1105-1118. <https://doi.org/10.1152/jn.00884.2013>

Chai, X. J., Castañán, A. N., Öngür, D., & Whitfield-Gabrieli, S. (2012). Anticorrelations in resting state networks without global signal regression. *NeuroImage*, *59*(2), 1420–1428. <https://doi.org/10.1016/j.neuroimage.2011.08.048>

Davey, J., Thompson, H. E., Hallam, G., Karapanagiotidis, T., Murphy, C., De Caso, I., ... & Jefferies, E. (2016). Exploring the role of the posterior middle temporal gyrus in semantic cognition: Integration of anterior temporal lobe with executive processes. *Neuroimage*, *137*, 165-177. <https://doi.org/10.1016/j.neuroimage.2016.05.051>

Fox, M. D., Snyder, A. Z., Vincent, J. L., Corbetta, M., Van Essen, D. C., & Raichle, M. E. (2005). The human brain is intrinsically organized into dynamic, anticorrelated functional networks. *Proceedings of the National Academy of Sciences*, *102*(27), 9673-9678. <https://doi.org/10.1073/pnas.0504136102>

Murphy, K., Birn, R. M., Handwerker, D. A., Jones, T. B., & Bandettini, P. A. (2009). The impact of global signal regression on resting state correlations: are anti-correlated networks introduced?. *Neuroimage*, *44*(3), 893-905. <https://doi.org/10.1016/j.neuroimage.2008.09.036>

Whitfield-Gabrieli, S., & Nieto-Castanon, A. (2012). Conn: A Functional Connectivity Toolbox for Correlated and Anticorrelated Brain Networks. *Brain Connectivity*, *2*(3), 125–141. <https://doi.org/10.1089/brain.2012.0073>
